# News and Events of the Week

**Published:** 1910-04-02

**Authors:** 


### News and Events of the Week.

The Edinburgh dinner of the Indian Medical Service will be held on Friday, May 27, 1910, at 7 p.m., at the Caledonian United Service Club, Edinburgh, Sir Alexander Christison, Bart., in the chair. Officers on the retired or active list wishing to be present at the dinner should communicate with Colonel Arnott, 8 Rothesay Place, Edinburgh.

The annual meeting of the Medical Mission Auxiliary will be held in the Queen's Hall, Langham Place, W., on Friday, May 6, at 7 p.m. The chair will be taken by Professor Carless, M.S., F.R.C.S.; the names of the speakers will be announced later. Tickets (including a few for reserved seats at 1s. each) may be obtained on application to the Superintendent, Loan Department, C.M. House.

According to Leewry, an aqueous extract of the common edible mushroom gives an intense violet colour-reaction with strong sulphuric acid. If only a trace of this be floated on the acid in a test-tube a marked violet colour zone will be evident, which disappears on warming. *Amanita phalloides*, the very poisonous fungus which is sometimes mistaken for the mushroom, gives a yellow colour with this test. A large number of other edible and poisonous fungi thus tested failed to give the violet colour. The reaction may be obtained with various sauces in which mushrooms are an ingredient, and it might be used to prove the identity of the dried mushrooms used on the Continent for culinary purposes.

At the Tyrone Assizes last week, the grand jury passed a resolution calling attention to the unrestricted sale of ether, and recommending that the drug be scheduled as a dangerous poison, and that chemists should be compelled to keep a register of the sale. Chief Baron Palles said he would have great pleasure in forwarding the resolution to the proper quarter, together with a strong recommendation that it be put into force. He was under the impression that at present there was, under an Act of Parliament, some restriction on the sale of ether, but, having regard to the evidence that had been given in one of the cases before the grand jury, he thought those regulations must be insufficient. At all events, he was happy to forward the resolution to the Under-Secretary, and he would have the matter looked into and everything done that was in his power to give effect to the recommendation, which he would have entered in the Crown book, and then send it to Sir James B. Dougherty for attention at the hands of the Government.

The Vice-Chancellor of the Manchester University (Dr. Hopkinson), speaking at a degree ceremony on the 23rd ult., announced the impending retirement of Mr. F. A. Southam, F.R.C.S., Professor of Clinical Surgery, and said that an intimation as to Mr. Southam's successor was expected shortly. In the medical school, he continued, they had somewhat remodelled the curriculum leading to the ordinary medical degree of the University. He thought those changes would benefit both the students and their future patients, as giving more opportunity for practical work. A special course in chemistry had been arranged for Manchester students who wished to use chemistry in their future work. As to the work after the stage when the student had obtained his degree or medical qualification, there were three branches of medical research to which special attention should be called. The first was what was called psychological medicine. At the next meeting of the Court in May a proposal would be made for the establishment of a special diploma in that subject, which was one of growing importance. He hoped the Court would favourably entertain the proposals drawn up by the Senate and Council. It would be a desirable thing if they could have one or two fellowships founded for research in special branches of medicine. He also hoped that very soon there would be developments in regard to medicine and hygiene as applied to certain industrial pursuits and trades. Results might be obtained which would be of enormous benefit in preventing the deterioration of the race in great industrial centres. He hoped they would receive the moral support of the public in this matter, and also of members of the medical profession who were in practice.

From the Native State of Hyderabad an urgent appeal has reached the Mission to Lepers on behalf of the many diseased outcasts among the population both of cities and villages. It is proposed to build an asylum near Nizamabad, within a radius of forty miles of which there are said to be 2,000 cases. Recently the bakers of the town were expelled, as the disease was rife among them, and the Mission doctors discovered lepers among the butchers. A local Hindu merchant has offered an excellent site and a substantial contribution towards the building. Donations for the erection and support of the asylum will be thankfully received by Mr. John Jackson (Organising Secretary), 33 Henrietta Street, Strand, W.C.

Gresham Lectures, 1910.—Four lectures on "Elementary Domestic Hygiene" will be delivered on Tuesday, April 5; Wednesday, April 6; Thursday, April 7; and Friday, April 8, 1910, by Dr. F. M. Sandwith, Gresham Professor of Physic. At the request of the Gresham Committee the lectures for the present term will be delivered at the City of London School, Victoria Embankment, E.C. (three minutes' walk from Blackfriars Bridge). The lectures are free to the public, and begin each evening at six o'clock.

The annual meeting of the Society for the Study of Inebriety will be held in the rooms of the Medical Society of London, 11 Chandos Street, Cavendish Square, W., on Tuesday, April 12, 1910, at 4 p.m. (afternoon meeting). A short address will be delivered by the President, after which a discussion on "Alcohol and Life Assurance" will be opened by Mr. William Bingham, J.P. Each member and associate is at liberty to introduce a visitor. Tea and coffee at 3.45; Council meeting at 3.30. The minimum annual subscription to the Society being a merely nominal one (5s., including copy of the *British Journal of Inebriety*, post free), the Council have resolved that a reserve fund be established to further the work of the Society, and every member and associate and all interested in the scientific study of alcoholism are earnestly invited to forward contributions to the Hon. Treasurer, G. Basil Price, M.D., Livingstone College, Leyton, N.E.

A death from hydrophobia was investigated by the Hackney Coroner last week. The deceased was a soldier who had been bitten by a rabid dog eight months ago at Gibraltar, and who had been treated at the Pasteur Institute at Paris.

The Third International Congress of School Hygiene will be held in Paris on August 2, under the honorary presidency of the Minister of Public Instruction. Applications for all information should be made to the general secretary, Dr. Dufestel, 10 Boulevard Magenta, Paris; and for information concerning the conditions for the voyages, lodgings, and excursions to the Agence Lubin, 36 Boulevard Haussmann, Paris.

A monument to the memory of the American dentist, Horace Wells, who was the first to introduce the practice of painless dentistry with the aid of nitrous-oxide gas, has been unveiled in Paris. The monument consists of a bust of Mr. Wells, supported by a white marble column, to which has been affixed a medallion of the physiologist, Paul Bert, who perfected the method of the American dentist.

The third number of "School Hygiene," a new monthly devoted to the subject which forms its title, is as useful and interesting as its predecessors. Dr. Clive Riviere contributes a sensible paper on heart strain in boys, which should be carefully read and studied by educationists and, let us add, by scoutmasters. Dr. Williams gives an account of the Sheffield open-air recovery school, and the other articles in the number deal with questions of general interest to doctors and schoolmasters.

A sessional meeting of the Royal Sanitary Institute will be held at 90 Buckingham Palace Road, on Wednesday, April 6, at 5 p.m., when a paper will be read on "Sanitary Aspects of Floods, and Measures to be taken to prevent Epidemics arising therefrom," by Dr. A. J. Martin, Inspecteur Général des Services d'Hygiène de la Ville de Paris. The chair will be taken at 5 p.m., by Mr. A. Wynter Blyth, Barrister-at-Law, M.R.C.S. Tickets for admission of visitors may be had on application to Mr. E. White Wallis, Secretary.

THE HOSPITAL. April 2, 1910.

